# Viral RNA in Blood as Indicator of Severe Outcome in Middle East Respiratory Syndrome Coronavirus Infection

**DOI:** 10.3201/eid2210.160218

**Published:** 2016-10

**Authors:** So Yeon Kim, Sun Jae Park, Sook Young Cho, Ran-hui Cha, Hyeon-Gun Jee, Gayeon Kim, Hyoung-Shik Shin, Yeonjae Kim, Yu Mi Jung, Jeong-Sun Yang, Sung Soon Kim, Sung Im Cho, Man Jin Kim, Jee-Soo Lee, Seung Jun Lee, Soo Hyun Seo, Sung Sup Park, Moon-Woo Seong

**Affiliations:** National Medical Center, Seoul, South Korea (S.Y. Kim, S. Park, S.Y. Cho, R. Cha, H.-G. Jee, G. Kim, H.-S. Shin, Y. Kim, Y.M. Jung);; Korea Centers for Disease Control and Prevention, Cheongju-si, South Korea (J.-S. Yang, S.S. Kim);; Seoul National University College of Medicine, Seoul (S.I. Cho, M.J. Kim, J.-S. Lee, S.J. Lee, S.H. Seo, S.S. Park, M.-W. Seong)

**Keywords:** Middle East respiratory syndrome, coronavirus, blood, prognosis, real-time PCR, viruses, MERS-CoV, zoonoses

## Abstract

We evaluated the diagnostic and clinical usefulness of blood specimens to detect Middle East respiratory syndrome coronavirus infection in 21 patients from the 2015 outbreak in South Korea. Viral RNA was detected in blood from 33% of patients at initial diagnosis, and the detection preceded a worse clinical course.

Middle East respiratory syndrome coronavirus (MERS-CoV) is a zoonotic, betacoronavirus lineage C RNA virus that was first identified in Saudi Arabia in 2012 (*1*). MERS-CoV causes respiratory and renal illness in humans, and infection often progresses to severe pneumonia, acute respiratory distress syndrome, renal failure, or death in a subset of patients (*2*). Risk factors, including patient age, preexisting health conditions, and high viral load in upper respiratory specimens, have been suggested to be related to disease severity and death (*3*,*4*). However, pathogenesis and clinical characteristics promoting recovery from infection or progression to serious organ failure have not been well elucidated.

Respiratory specimens are preferred for viral RNA detection and confirmatory diagnosis of MERS-CoV infection in humans (*5*). MERS-CoV has broad tissue tropism, including the kidney, intestinal tract, liver, histiocytes, macrophages, and T lymphocytes, but viral RNA has been found inconsistently in blood, urine, and fecal specimens (*6*–*10*). Reports have described small numbers of cases with extrapulmonary virus; therefore, it remains unclear whether extrapulmonary specimens have any diagnostic usefulness in determining infection or whether extrapulmonary viral detection has clinical implications in disease management.

A large MERS-CoV outbreak occurred in 2015 in South Korea. This outbreak comprised the first imported case and subsequent infection of 185 patients (*11*). Our study aimed to evaluate the diagnostic utility of blood specimens for MERS-CoV infection by using large numbers of patients with a single viral origin and to determine the relationship between blood viral detection and clinical characteristics.

## The Study

We collected 21 pairs of EDTA whole blood and serum specimens from 21 patients with MERS-CoV after admission to the National Medical Center in Seoul, South Korea. MERS-CoV infection initially was diagnosed by the Korea Centers for Disease Control and Prevention using respiratory specimens (*11*). After admission, each patient was reassessed for epidemiologic information and clinically managed with monitoring.

Specimens were stored at −80°C before analyses. Viral RNA was extracted and eluted with a MagNA Pure LC 2.0 automated nucleic acid extractor and MagNA Pure LC total nucleic acid isolation kit (both from Roche Diagnostics, Mannheim, Germany) according to the manufacturer’s instructions. Specimen volumes were 100 μL EDTA whole blood and 200 μL serum, and elution volumes were 100 μL for EDTA whole blood and 50 μL for serum. One-step, real-time reverse transcription PCR (rRT-PCR) was performed for the 3 MERS-CoV gene regions (upstream envelope [upE], open reading frame [ORF] 1a, and nucleocapsid) with an AgPath-ID One-step RT-PCR kit (Applied Biosystems, Foster City, CA, USA) and an ABI7500 real-time PCR system (Applied Biosystems). Human ribonuclease (RNase) P was amplified in parallel for sample quality control (*5*,*12*,*13*). In each test, the viral RNA was considered detected when amplification before cutoff was observed from at least 2 different targets in MERS-CoV with pass of sample quality control. The robustness of rRT-PCR was demonstrated for qualitative concordance of positivity or negativity by using different types of specimens (Technical Appendix Table 1). We calculated the viral copy concentration in the blood using standard curves constructed from the cycle threshold (C_t_) of serially diluted 10^5^ copies/μL of upE RNA (provided by the University of Bonn Medical Center, Bonn, Germany).

The results of the blood viral RNA analyses did not affect clinical management. We assessed the relationships among clinical and molecular diagnostic factors with the IBM SPSS Statistics program 22.0 (SPSS Inc., Chicago, IL, USA). In each test, p<0.05 was considered statistically significant. The institutional review board of the National Medical Center approved this study (H-1508-057-002).

We assessed patient demographics, their clinical features, and disease outcomes (Tables 1, 2; Technical Appendix Table 2). The time difference was an average of 1.5 days (median 1, range 0–5 days) between when the initial diagnostic respiratory specimens and blood specimens were obtained. At admission, viral RNA was detected in 6 (29%) of 21 EDTA whole blood and 6 (29%) of 21 serum samples from infected patients. Two patients showed viral positivity in either specimen subtype of EDTA whole blood or serum; therefore, the overall detection rate for MERS-CoV was 33% (7/21) in blood. The concordance rate of viral assay was 90% (19/21) between EDTA whole blood and serum specimens. Blood virus concentration was 3,130 copies/mL EDTA whole blood (range 2,080–17,400 copies/mL), equivalent to a median upE C_t_ of 37.55, and 1,300 copies/mL serum (range 490–10,200 copies/mL, median upE C_t_ 36.88).

**Table 1:** Demographic characteristics of 21 Middle East respiratory syndrome coronavirus–infected patients, South Korea, 2015

Characteristic	Value
No.	21
Median age, y (range)	64 (23–86)
Sex, no.	
M	9
F	12
Median hospitalization, d (range)	17 (2–138)
Death rate, %	23.8
Median no. days exposed to virus (range)*	3 (1–20)
Median duration between symptom onset and initial diagnosis, d (range)	2 (0–12)†

*Based on contact history. †Two asymptomatic patients were excluded from analysis.

**Table 2:** Differences in Middle East respiratory syndrome coronavirus detection by rRT-PCR among specimen types, South Korea, 2015*

Characteristic	Respiratory specimen type	Blood specimen type	
EDTA whole blood	Serum
No. specimens	20 sputum; 1 endotracheal aspirate	21	21
Days from initial confirmatory diagnosis using respiratory specimens to blood sampling	Set as day 0	1.4 (median 1, range 0–5)	1.5 (median 1, range 0–5)
Viral gene region rRT-PCR			
Upstream of E			
Total positive results/total tests (%)	21/21 (100)	6/21 (29)	6/21 (29)
Median C_t_ (range)	28.48 (19.70–33.46)†	37.55 (35.34–38.07)	36.88 (34.24–38.14)
Open reading frame 1a			
Total positive results/total tests (%)	21/21 (100)	0/21 (0)	0/21 (0)
Median C_t_ (range)	29.28 (21.00–34.21)†	Not detected	Not detected
Nucleocapsid			
Total positive results/total tests (%)	Not performed	7/21 (33)	7/21 (33)
Median C_t_ (range)	Not performed	36.37 (35.62–38.04)	34.62 (32.93–38.84)

*C_t_, cycle threshold; rRT-PCR, real-time reverse transcription PCR. †One patient whose C_t_ was not reported was excluded.

Blood viral RNA positivity at admission was associated with fever >37.5°C on the sampling date (p = 0.007), requirement for mechanical ventilation during the following clinical course (p = 0.003) and extracorporeal membrane oxygenation (p = 0.025), and patient death (p = 0.025, all by 2-tailed Fisher exact test; Figure). Blood viral RNA positivity was not associated with viral C_t_ in the initial diagnostic lower respiratory specimens, or requirement of oxygen supplementation during the following clinical course. Between the blood viral RNA-positive and -negative groups, we found no differences in age, duration from symptom onset to diagnosis of MERS-CoV infection, or an invasive procedure before the specimens were obtained (Technical Appendix Table 3).

Viral loads in the lower respiratory specimens at the initial confirmatory diagnosis showed no effect on patient survival (Figure). Patient death was not associated with length of time from symptom onset to diagnosis of MERS-CoV infection (Technical Appendix Table 3).

**Figure:**
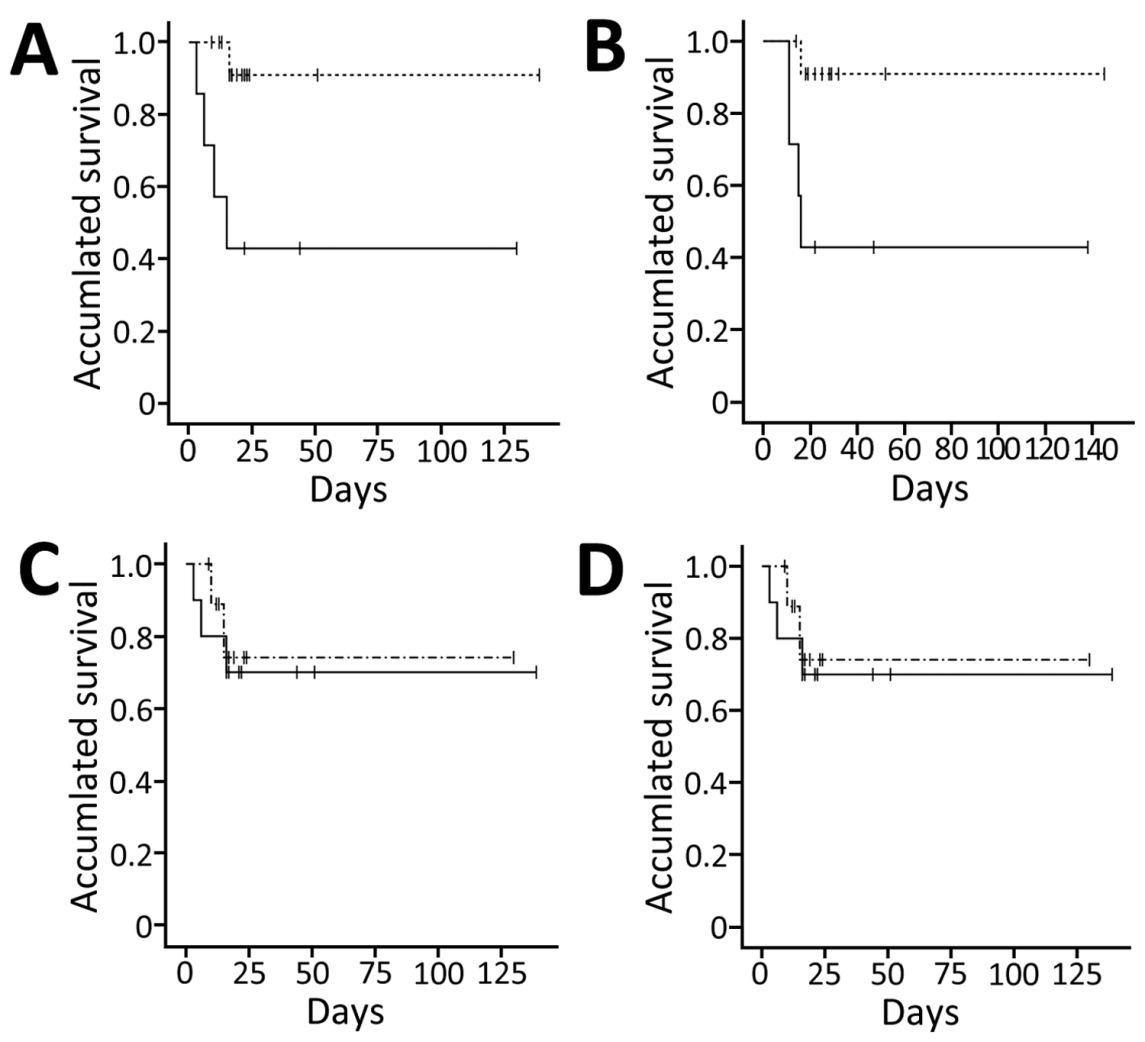
Differences in survival among Middle East respiratory syndrome coronavirus–infected patients, South Korea, 2015. A, B) Survival difference between the blood viral RNA-positive (solid line) and -negative (broken line) groups. Survival was defined as the time from initial confirmatory diagnosis to death before hospital discharge (A) (Kaplan-Meier survival analysis, log rank p = 0.009; Breslow p = 0.006) and as the time from symptom onset to death (B) (Kaplan-Meier survival analysis, log rank p = 0.017; Breslow p = 0.015). C, D) Survival difference between the high respiratory viral load (solid line) and low respiratory viral load (broken line) groups. Viral loads were classified into 2 groups: patients who harbored viral loads above the median load of patients and patients who harbored below. Survival was defined as time from initial confirmatory diagnosis to death. Cycle threshold (C_t_) values were calculated for real-time reverse transcription PCRs targeting the upstream of envelope region (C) and open reading frame 1a region (D) (Kaplan-Meier survival analysis, log rank p = 0.739; Breslow p = 0.630). Tick marks along data lines indicate data-censored time points.

Our results showed that the detection rate of blood viral RNA was low in the early phase of infection in patients with a confirmed diagnosis, similar to results from a previous study (*14*). These findings contrasted with those of severe acute respiratory syndrome coronavirus infection (*15*). Therefore, in the case of MERS-CoV infection, blood does not have the highest diagnostic yield for the initial confirmatory diagnosis. The viral load in blood was low, even in detected cases. A proportion of MERS-CoV isolates in the 2015 Korea outbreak harbored a C→T substitution in the third nucleotide of the ORF1a primer binding site (GenBank accession nos. KT374052–374055). This mismatch may partially contribute to the insensitivity of ORF1a assay observed in this study. An alternative sensitive target replacing ORF1a might be useful in studies using blood specimens.

Blood viral RNA has been detected in a few case reports of MERS-CoV fatalities (*8*–*10*). Our data of 42 specimens from cross-sectional time points focusing on early viremia showed that blood viral RNA was present in a subpopulation of patients and that these patients had significantly poorer prognoses, as demonstrated by the need for more frequent mechanical ventilation and the increased risk for death. Further large studies using serial daily specimens that are collected throughout the admission period–both upper and lower respiratory specimens and paired measurement of viral RNA and antibody in blood–might help overcome the limitation of the current study, which included relatively small numbers of deceased patients (24% [5/21]).

## Conclusions

Our data showed a detection rate of 33% for viral RNA in blood at initial diagnosis, which was insufficient for initial confirmatory diagnosis. Blood viral RNA at the early phase was related to a worse clinical course in infected patients and might be a good prognostic indicator of severe outcome. Measuring blood viral RNA at hospital admission might be useful.

Technical Appendix Sensitivity, reproducibility, and inhibitory effect among different specimen types for the Middle Ease respiratory syndrome coronavirus (MERS-CoV) real-time reverse transcription PCR; viral RNA analyses of MERS-CoV–infected patients; and statistical relationships among clinical and molecular diagnostic factors.
