# Supplementary material for: Viral RNA in Blood as Indicator of Severe Outcome in Middle East Respiratory Syndrome Coronavirus Infection
Source: Emerg Infect Dis. 2016 Oct;22(10):1813–6. doi: 10.3201/eid2210.160218 (PMC5038397; doi:10.3201/eid2210.160218)
Supplement: Technical Appendix — Sensitivity, reproducibility, and inhibitory effect among different specimen types for the Middle Ease respiratory syndrome coronavirus (MERS-CoV) real-time reverse transcription PCR; viral RNA analyses of MERS-CoV–infected patients; and statistical relationships among clinical and molecular diagnostic factors. [file 16-0218-Techapp-s1.pdf]

Article DOI: http://dx.doi.org/10.3201/eid2210.160218

Viral RNA in Blood as Indicator of Severe Outcome in Middle East Respiratory Syndrome Coronavirus Infection

Technical Appendix

Technical Appendix Table 1. Sensitivity, reproducibility, and inhibitory effect among different specimen types for the Middle East respiratory syndrome coronavirus rRT-PCR*

	upE RNA concentration, copies/μL RNA				
Specimen,† C_t	1,000	100	10	1	0
Water, Inhibitor-negative control					
Test 1	30.24	32.93	36.54	42.32	ND
Test 2	29.86	33.45	36.31	ND	Not tested
Test 3	30.34	33.45	36.68	ND	ND
Test 4	29.91	33.40	37.44	ND	Not tested
Mean C_t	30.09	33.31	36.74	42.32	ND
Total positive results/total tests	4/4	4/4	4/4	1/4	0/2
0.01% Sodium dodecyl sulfate, Inhibitor-positive control					
Test 1	33.77	36.26	38.36	ND	ND
Test 2	33.44	35.97	41.85	ND	Not tested
Test 3	33.67	37.09	ND	ND	ND
Test 4	33.61	37.23	44.90	ND	Not tested
Mean C_t	33.62	36.64	41.70	ND	ND
Total positive results/total tests	4/4	4/4	3/4	0/4	0/2
RNA extracted from frozen EDTA whole blood					
Test 1	30.66	33.81	36.45	40.19	ND
Test 2	30.60	34.22	37.73	39.44	Not tested
Mean C_t	30.63	34.02	37.09	39.82	ND
Total positive results/total tests	2/2	2/2	2/2	2/2	0/1
RNA extracted from unfrozen EDTA whole blood					
Test 1	30.82	34.47	39.44	ND	ND
Test 2	31.32	34.69	38.56	ND	Not tested
Mean C_t	31.07	34.58	39.00	ND	ND
Total positive results/total tests	2/2	2/2	2/2	0/2	0/1
RNA extracted from plasma of EDTA whole blood					
Test 1	30.37	34.25	36.65	ND	ND
Test 2	30.67	34.79	38.20	ND	Not tested
Mean C_t	30.52	34.52	37.43	ND	ND
Total positive results/total tests	2/2	2/2	2/2	0/2	0/1
RNA extracted from serum of clotted whole blood					
Test 1	30.97	33.57	36.51	ND	ND
Test 2	30.92	35.20	36.67	ND	Not tested
Mean C_t	30.95	34.39	36.59	ND	ND
Total positive results/total tests	2/2	2/2	2/2	0/2	0/1

*C_t, cycle threshold; ND, not detected; rRT-PCR, real-time reverse transcription PCR.
†To evaluate the inhibitory effect in RNA from each specimens, the *upE* RNA with known copy numbers was spiked in series of quadruplicated controls and duplicated tests to generate panels of 5 concentrations (1,000, 100, 10, 1, and 0 copies/μL), and the rRT-PCR *upE* was performed afterward.

Technical Appendix Table 2. Viral RNA analyses of Middle East respiratory syndrome coronavirus–infected patients: demographic and clinical features, and disease outcomes, South Korea, 2015*

Case identifier	rRT-PCR: Using EDTA whole blood: upE† (C_t)	rRT-PCR: Using EDTA whole blood: N‡ (C_t)	rRT-PCR: Using serum: upE (C_t)	rRT-PCR: Using serum: N (C_t)	Virus detection in blood, EDTA whole blood, and/or serum	Outcome	Patient sex/age, y	No. days exposed to virus, based on contact history	Days from symptom onset to initial diagnosis using respiratory specimen	Days from initial diagnosis to specimen sampling: EDTA whole blood	Days from initial diagnosis to specimen sampling: Serum	Respiratory specimen: C_t from rRT-PCR using initial specimen: upE	Respiratory specimen: C_t from rRT-PCR using initial specimen: ORF1a	Specimen subtype	Hospitalized, d
01	Positive (35.34)	Positive (36.37)	Positive (37.32)	Positive (34.62)	Positive	Died	M/71	6	5	1	1	23.45	24.30	Sputum	5
02	Positive (37.81)	Positive (36.35)	Positive (37.63)	Positive (34.74)	Positive	Died	M/56	3	1	5	5	28.56	31.00	Sputum	10
03	Positive (37.38)	Positive (35.81)	Positive (36.38)	Positive (33.78)	Positive	Died	M/86	7	1	1	3	33.19	30.83	Sputum	10
04	Positive (38.07)	Positive (36.70)	Not detected	Positive (35.76)	Positive	Died	F/70	1	12	2	2	19.70	21.00	Sputum	2
05	Positive (37.46)	Positive (36.40)	Positive (36.43)	Positive (34.00)	Positive	Survived	M/68	20	8	1	1	29.35	31.45	Endotracheal aspirate	129
06	Positive (37.63)	Positive (35.62)	Positive (34.24)	Positive (32.93)	Positive	Survived	M/71	1	3	1	1	23.34	23.01	Sputum	43
07	Not detected	Not detected	Positive (38.14)	Positive (38.84)	Positive	Survived	F/75	3	0	3	1	Positive, value not reported	Positive, value not reported	Sputum	21
08	Not detected	Positive (38.04)	Not detected	Not detected	Not detected	Survived	M/79	2	6	1	1	24.44	25.89	Sputum	138
09	Not detected	Not detected	Not detected	Not detected	Not detected	Died	M/76	1	0	1	1	28.10	28.65	Sputum	16
10	Not detected	Not detected	Not detected	Not detected	Not detected	Survived	F/63	6	2	0	0	26.23	26.63	Sputum	17
11	Not detected	Not detected	Not detected	Not detected	Not detected	Survived	F/46	1	2	0	0	29.30	30.45	Sputum	23
12	Not detected	Not detected	Not detected	Not detected	Not detected	Survived	F/39	3	10	4	4	24.88	25.44	Sputum	18
13	Not detected	Not detected	Not detected	Not detected	Not detected	Survived	F/23	3	9	2	2	28.40	28.04	Sputum	14
14	Not detected	Not detected	Not detected	Not detected	Not detected	Survived	F/64	3	2	0	0	30.60	29.70	Sputum	12
15	Not detected	Not detected	Not detected	Not detected	Not detected	Survived	M/86	7	1	1	2	20.14	22.20	Sputum	20
16	Not detected	Not detected	Not detected	Not detected	Not detected	Survived	F/83	7	10	1	1	33.22	34.21	Sputum	18
17	Not detected	Not detected	Not detected	Not detected	Not detected	Survived	F/52	5	1	1	1	28.40	28.85	Sputum	50
18	Not detected	Not detected	Not detected	Not detected	Not detected	Survived	M/50	1	4	2	2	32.15	32.58	Sputum	24
19	Not detected	Not detected	Not detected	Not detected	Not detected	Survived	F/24	3	1	2	2	33.46	33.12	Sputum	15
20	Not detected	Not detected	Not detected	Not detected	Not detected	Survived	F/24	12	Asymptomatic	1	1	32.79	32.51	Sputum	8
21	Not detected	Not detected	Not detected	Not detected	Not detected	Survived	F/24	7	Asymptomatic	0	0	32.59	32.26	Sputum	13

*C_t, cycle threshold; rRT-PCR, real-time reverse transcription PCR.
†rRT-PCR *upE* was originally developed in reference *12*.
‡rRT-PCR *N2* was originally developed in Reference *13*.

Technical Appendix Table 3. Statistical relationships among clinical and molecular diagnostic factors associated with MERS-CoV infection, South Korea, 2015

Association between factors	p value	Statistical test
Blood viral RNA positivity and viral C_t in initial diagnostic lower respiratory specimens	0.312	Mann-Whitney U
Blood viral RNA positivity and requirement of oxygen supplementation during the following clinical course	0.061	2-Tailed Fisher exact
Differences in age between the blood viral RNA-positive and -negative groups	0.094	Mann-Whitney U
Differences in duration from symptom onset to diagnosis of MERS-CoV infection between the blood viral RNA-positive and negative groups	0.967	Mann-Whitney U
Differences in the experience of invasive procedure before the specimens were obtained between the blood viral RNA-positive and -negative groups	0.337	2-Tailed Fisher exact
Patient death and the length of time from symptom onset to diagnosis of MERS-CoV infection	0.559	Mann-Whitney U

*C_t, cycle threshold; MERS-CoV, Middle East respiratory syndrome coronavirus.
